# Translocation of the thioesterase domain for the redesign of plipastatin synthetase

**DOI:** 10.1038/srep38467

**Published:** 2016-12-23

**Authors:** Ling Gao, Hongxia Liu, Zhi Ma, Jinzhi Han, Zhaoxin Lu, Chen Dai, Fengxia Lv, Xiaomei Bie

**Affiliations:** 1 College of Food Science and Technology, Nanjing Agricultural University, Key Laboratory of Food Processing and Quality Control, Ministry of Agriculture of China, 1 Weigang Nanjing 210095, P.R. China; 2 College of Life Science, Nanjing Agricultural University, Ministry of Agriculture of China, 1 Weigang Nanjing 210095, P.R. China

## Abstract

Non-ribosomal peptide synthetases (NRPSs) are large enzymatic complexes that catalyse the synthesis of biologically active peptides in microorganisms. Genetic engineering has recently been applied to reprogram NRPSs to produce lipopeptides with a new sequence. The carboxyl-terminal thioesterase (TE) domains from NRPSs catalyse cleavage products by hydrolysis or complex macrocyclization. In this study, we modified plipastatin synthetase by moving the intrinsic TE region to the end of the internal thiolation (T) domains, thus generating *Bacillus subtilis* strains that could produce new truncated cyclic or linear peptides of the predicted sequence, which further provided an important insight into the regioselectivity of plipastatin TE. The TE was capable of recognizing and catalysing the lactone formation between _L_-Try_3_ with the last few residues _L_-Pro7 and _L_-Gln8 at the C-terminus. Additionally, the unmatched linkers connecting the TE region and T domain resulted in nonproduction strains, suggesting that the native T–TE linker is necessary and sufficient for the TE domain to release the products from the hybrid enzymes. This is the first report to demonstrate truncated cyclic lipopeptides production and module skipping by simply moving the TE domain forward in an NRPS system.

A vast number of biologically active natural peptides are produced in a template-directed manner using multimodular enzymes known as non-ribosomal peptide synthetases (NRPSs)^1^,^2^. Surfactants (e.g. surfactin), fungicides (e.g. fengycin/plipastatin, iturin), and prominent drugs (e.g. cyclosporin A, daptomycin)^3^,^4^ are examples of commonly used peptides derived from NRPSs. These lipopeptides have a common feature of microcyclization, which decreases the structural flexibility of the peptide, thus constraining it into the biologically active conformation^5^. Moreover, these lipopeptides contain some unusual residues such as non-proteinogenic amino acids, d-amino acids, N- and C-methylated residues, and glycocylated residues, as well as phosphorylated residues, which has led to their enormous structural diversity.

Plipastatin, an antifungal lipopeptide antibiotic, was originally isolated from *Bacillus cereus* BMG302-fF67 as an inhibitor of phospholipase A2^6^ and contains a cyclic peptide of 10 amino acids with a β-hydroxy fatty acid chain (C14 to C18) attached to the N-terminus of the peptide (Fig. 1). Previous studies have established that plipastatin is assembled on five giant NRPS multi-enzymes, PPSA, PPSB, PPSC, PPSD, and PPSE^7^,^8^, encoded by the *ppsA*, *ppsB*, *ppsC*, *ppsD*, and *ppsE* genes, respectively, in the plipastatin synthetase operon (Fig. 1). According to their biosynthetic function, the NRPSs can be subdivided into distinct modules responsible for initiation, elongation, and termination. Each module has three domains: a condensation (C) domain, responsible for peptide bond formation; an adenylation (A) domain, which recognizes and activates the substrate amino acids; and a thiolation (T) domain or peptidyl carrier protein (PCP), which tethers the activated substrates and the growing peptide chain. Some of the modules also have epimerase (E) domains that convert l-amino acids to their d-isomers. The terminal modules of NRPSs often have a thioesterase (TE) domain that is involved in cyclization and product release. The sequence of the peptide product directly corresponds to the linear arrangement of modules and domains within the biosynthetic template. This structure enables the development of straightforward strategies for the rational design of NRPSs via the recombination of gene fragments to generate arrays of peptide derivatives and thus expand the diversity of microbial-produced lipopeptides.

In recent years, engineering of deletions, insertions, and module exchange in NRPS systems has proven to be a useful strategy to obtain lipopeptides of a targeted sequence^3^,^9^,^10^,^11^,^12^. Specifically, many daptomycin derivatives have been produced by the replacement of a complete catalytic unit, and some of these daptomycin-related lipopeptides have even shown improved activity compared to the native antibiotic^13^,^14^. The *in vitro* characterization of various TE domains has been reported based on purified proteins^15^,^16^,^17^,^18^. However, *in vivo* studies on the TE domain of plipastatin synthetase using molecular engineering strategies are scarce. Valuable biologically active compounds synthesized by an NRPS system in hosts could be better suited for industrial production. Here, we moved the TE domain of plipastatin synthetase forward, and evaluated the effect of this change on the production of truncated active analogues *in vivo*. We further investigated the structure of the novel peptides generated to analyse the catalytic activity and selectivity of the TE domain in the new hybrid enzymes.

## Methods

### Strains, culture conditions, and homologous recombination of plasmids

The bacterial strains and plasmids used in this study are listed in Table 1. *Bacillus subtilis* was grown in Luria-Bertani (LB) medium at 37 °C. Landy fermentation medium buffered with 0.1 M 3-(N-morpholino) propanesulfonic acid (MOPS) was used as described previously^19^. Plasmids were transferred from *Escherichia coli* JM110 into *Bacillus subtilis* pB2-L by natural competence, as previously described^20^. The transformants were selected for kanamycin resistance at 20 μg/mL on LB agar. The recombinant clones were selected by a two-step replacement recombination procedure as described previously^21^. Briefly, in the first step, a *Bacillus* strain bearing an integrative plasmid that contained a gene replacement construct was grown in LB medium at 37 °C (a non-permissive temperature for plasmid replication). The entire plasmid was inserted into the chromosome via a single crossover between the target gene and a homologous sequence on the plasmid. In the second step, a separate clone of the integrant was cultured in LB medium at 30 °C for 48 h to induce the second exchange event and excise the plasmid. Kanamycin-sensitive (Kan^S^) clones with either the parental or mutant sequence were obtained and verified by PCR analysis and sequencing. The kanamycin resistance gene was not amplified, but the upstream and downstream regions of the homologous sequence could be amplified, and then sequencing confirmed the mutant sequence.

### Plasmid construction

The integrative plasmids that were used to move the TE domain forward to the end of the T domain were all derivatives of pKS2^22^. For the construction of plasmids pKS-7ProTTE, pKS-8GlnTTE, and pKS-9TyrTTE, gene fragments encoding the seventh T domain, eighth T domain (harbouring the native T-C linker), ninth T domain (harbouring the native T-E linker), and the TE domain of plipastatin synthetase were amplified using strain pB2-L chromosomal DNA as a template and the primers 7ProTlinker-F/R, 8GlnTlinker-F/R, 9TyrTlinker-F/R, and TE-F/R, respectively. All primers are listed in Supplemental Table S1. The 7ProTlinker-TE, 8GlnTlinker-TE, and 9TyrTlinker-TE fusions were constructed by splice overlap extension polymerase chain reaction (SOE-PCR) using the 7ProTlinker, 8GlnTlinker, and TE fragment as the template, and the primers 7ProTlinker-F/S7-R and S7-F/TE-R, 8GlnTlinker-F/S8-R and S8-F/TE-R, and 9TyrTlinker-F/S9-R and S9-F/TE-R, respectively. Then, the fused fragments 7ProTlinker-TE, 8GlnTlinker-TE, and 9TyrTlinker-TE were respectively inserted into the restriction endonuclease sites *Sal*I and *Kpn*I of pKS2.

The plasmids pKS-7ProTE_Long_, pKS-8GlnTE_Long_, and pKS-9TyrTE_Long_ were constructed as follows. Gene fragments encoding the seventh T domain, eighth T domain, ninth T domain, and TE_Long_ region (harbouring the native T-TE linker) of plipastatin synthetase were amplified by PCR from chromosomal DNA of strain pB2-L using pfu DNA polymerase (Fermentas, MA, USA) with the primers 7ProT-F/R, 8GlnT-F/R, 9TyrT-F/R, and TE_Long_-F/R, respectively. The fused fragments 7ProTTE_Long_, 8GlnTTE_Long_, and 9TyrTTE_Long_ were constructed by SOE-PCR using the primers 7ProT-F/P7-R and P7-F/TE_Long_-R, 8GlnT-F/P8-R and P8-F/TE_Long_-R, and 9TyrT-F/P9-R and P9-F/TE_Long_-R, respectively, which were then inserted into the restriction endonuclease sites *Sal*I and *Kpn*I of pKS2. All inserted fragments were confirmed by sequencing (Genscript, Nanjing, China).

### Lipopeptides production, purification, and identification

The recombinant strains were fermented in triplicate for 3 days at 33 °C, 180 rpm. Cultures were centrifuged at 5000× *g* for 15 min at 4 °C to remove the bacterial cells. The supernatant was adjusted to pH 2.0 with 6 N HCl to precipitate plipastatin and plipastatin-derived peptides. The pellet was then collected and extracted with methanol. The crude extract was filtered through a 0.22-μm filter and analysed by liquid chromatography-electrospray ionization-mass spectrometry (LC-ESI-MS) using an LTQ Orbitrap XL hybrid mass spectrometer (Thermo Scientific, USA). Five microliters of methanolic extract was loaded onto a C18 reverse-phase column (Agilent, 2.1 × 100 mm, 3 μm particle size). The separation of lipopeptides was achieved using acetonitrile/H_2_O/0.2% formic acid as the mobile phase. The flow rate was maintained at 0.2 mL/min with a gradient of 22 min of 5% vol/vol acetonitrile for 2 min, 5–95% vol/vol acetonitrile for 13 min, 95% vol/vol acetonitrile for 3 min, and 95–5% vol/vol acetonitrile for 3 min. The elution was monitored by a total ion chromatogram at 205 nm. MS spectra were recorded in positive ion mode within a mass range of 300–1500 m/z. The molecular ions were further characterized by LTQ Orbitrap tandem mass spectrometry (MS/MS) using the following parameters: ion spray voltage at 2.5 kV, sheath gas at 20 units, capillary temperature at 300 °C, capillary voltage at 41 V, and tube lens at 110 V. Xcalibur software was used for visualization of the high-resolution spectral profile data (Thermo Fischer Scientific, Inc., 2nd Edition SP2).

## Results

### TE domain integration into the NRPS subunit PPSD

In previous experiments, we found that deletion of the TE domain of plipastatin synthetase results in the synthesis of truncated enzymes that were unable to produce the corresponding peptides (data not shown). This result suggested that the TE domain is necessary for plipastatin production, which is consistent with a study on the surfactin TE domain reported by de Ferra *et al*.^23^. Therefore, in the present study, we examined the productivity of truncated peptide synthetase after fusion of the TE domain to the end of the T domain in subunit PPSD.

**Figure 1:**
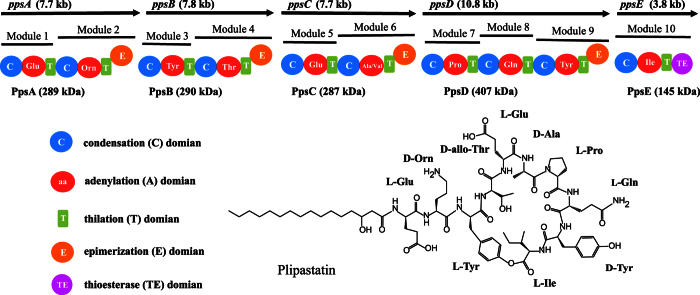
Structure of plipastatin (PubChem CID:102466606) and schematic diagram of the plipastatin biosynthesis operon (*pps*). Five NRPSs are encoded by the genes *ppsABCDE*. Based on their function, the five distinct synthetases, PpsA–E, can be divided into modules and domains. Each module is comprised of condensation (C), adenylation (A), thiolation (T), and epimerization (E) domains that are responsible for the activation, attachment, and modification of one constitutive amino acid residue. At module 10, a terminal thioesterase (TE) domain catalyses linear peptide cyclization and releases the final product, plipastatin.

**Figure 2:**
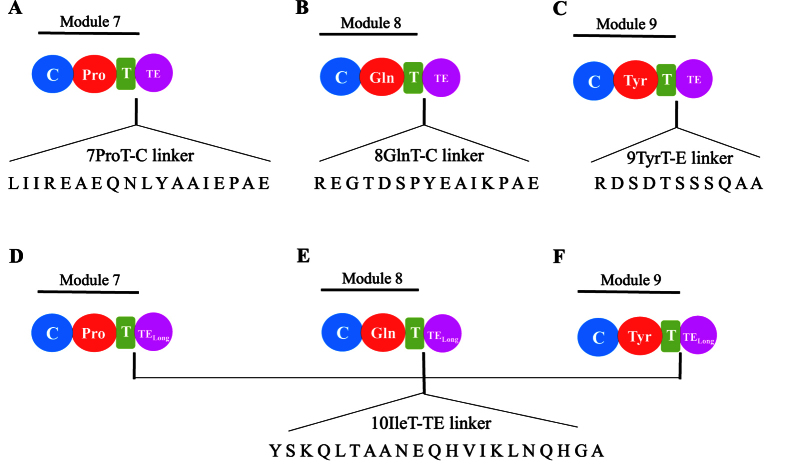
The linkers connecting the thioesterase (TE) domain with various thiolation (T) domains in new hybrid non-ribosomal peptide synthetases. The 7ProT-C, 8GlnT-C, and 9TyrT-E linkers were connected to the TE domain, respectively (**A**–**C**). TE_Long_, containing the native 10IleT-TE linker, was connecting to different T domains of the PPSD subunit (**D**–**F**). 7ProT-C: linker region between the T domain of module 7 and the condensation (**C**) domain of module 8. 8GlnT-C: linker region between the T domain of module 8 and the C domain of module 9. 9TyrT-E: linker region between the T and epimerization (**E**) domains in module 9.

In this experiment, the TE domain (25 kDa, 212 residues) was respectively integrated into the end of the seventh, eighth, and ninth T domains in plipastatin NRPSs (Fig. 1). In describing the current experiments, we present the inter-domain linker regions as hyphens. Different linkers were used to connect the TE domains in the different constructs. The 7ProT-TE and 8GlnT-TE linkers were consistent with the sequences of the 7ProT-C linker (Fig. 2A) and 8GlnT-C linker (Fig. 2B), respectively, whereas the 9TyrT-TE linker was derived from the linker region between the T and E domains in subunit PPSD (Fig. 2C). The integrative plasmids containing the appropriate constructs were transferred into *B. subtilis* pB2-L, and then integrations of the TE domain were generated using a two-step replacement procedure, as described previously^21^. Use of this two-step method could avoid the potential impact of an exogenous antibiotic resistance gene. Colony PCR analysis and sequencing was used to examine the presence of the desired recombinant clone and confirm that the later part of NRPSs of plipastatin from inserted TE-domain is completely removed from genome to prevent unexpected recombination. The three recombinants (LP1, LP2, LP3; Table 1) were unable to produce any lipopeptide related to plipastatin, which suggested that the hybrid synthetases were inactive. According to these results, we speculated that none of the linkers connecting the TE domain matched, resulting in loss of function of the TE domain in the hybrid peptide synthetase.

### TE_Long_ domain integration into the NRPS subunit PPSD and the role of the native T-TE linker

**Figure 3:**
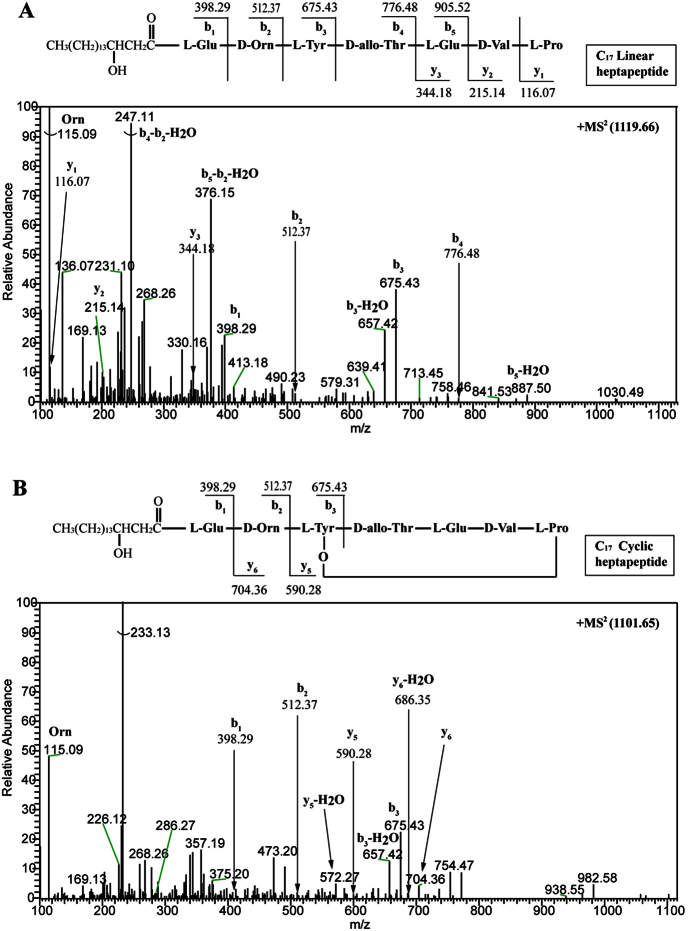
Liquid chromatography-electrospray ionization-tandem mass spectra of [M + H]^+^ ions of the linear heptapeptide at 1119.65 m/z (**A**) and the cyclic heptapeptide at 1101.65 m/z (**B**).

**Figure 4:**
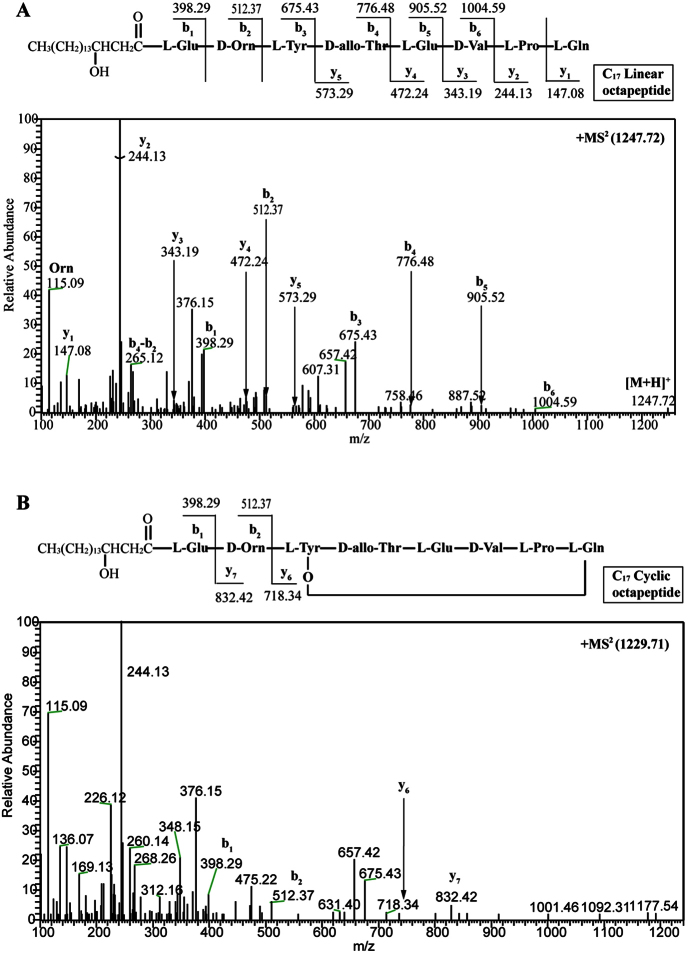
Liquid chromatography-electrospray ionization-tandem mass spectra of [M + H]^+^ ions of the linear octapeptide at 1247.72 m/z (**A**) and the cyclic octapeptide at 1229.71 m/z (**B**).

**Figure 5:**
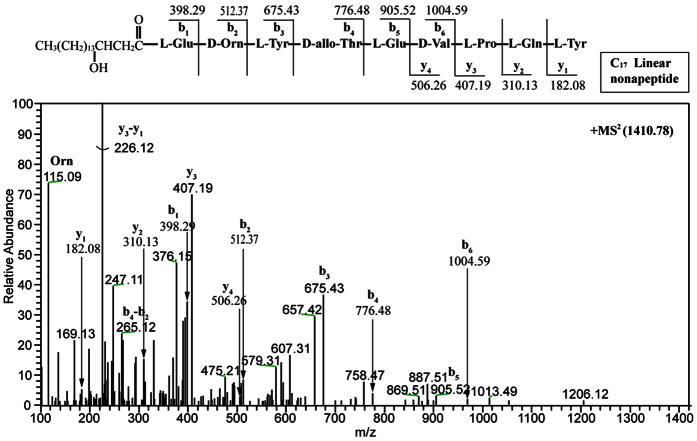
Liquid chromatography-electrospray ionization-tandem mass spectra of [M + H]^+^ ions of the linear nonapeptide at 1410.78 m/z.

To validate our speculation, we maintained the native 10IleT-TE linker in the hybrid synthetases. The TE_Long_ (28 kDa, 233 residues) domain, containing a linker region between the 10IleT and TE domains in subunit PPSE, was fused to the end of the T domain that originated from 7Pro-, 8Gln-, and 9Tyr-activating modules, respectively. In the hybrid peptide synthetases, the TE domain was connected with the native T-TE linker, as shown in Fig. 2D–F. The recombinant strains (LP4, LP5, and LP6; Table 1) produced the predicted cyclic or linear lipopeptides, as determined by high-resolution LC-ESI-MS. Table 2 summarizes the data for the major [M + H]^+^ peaks obtained from the different plipastatin derivatives. Comparison of these data with the molecular weight of plipastatin clearly indicated the fused position of the TE_Long_ domain, as deduced from the expected sequences of these truncated lipopeptides. Linear heptapeptides, octapeptides, and nonapeptides were respectively detected in strains LP4, LP5, and LP6 as expected, and the linear structure was further confirmed by MS/MS analysis (Figs 3A, 4A and 5A). For example, in the LC-ESI-MS/MS spectrum of the [M + H]^+^ ions at 1119.66 m/z (Fig. 3A), the observed b- and y-fragment ions permitted coverage of the entire sequence, confirming that the amino acid composition of the putative linear heptapeptide was Glu-Orn-Tyr-Thr-Glu-Val-Pro, where the β-OH fatty acid containing 17 carbons was linked to the N-terminus of the peptide. The [M + H]^+^ ions at 1105.6, 1119.6, 1133.7, and 1147.7 m/z differed in mass by multiples of 14 Da, indicating that the linear heptapeptides with identical peptide sequences varied with respect to the chain length of the β-OH fatty acid (C_16_, C_17_, C_18_, and C_19_, respectively). Similarly, Figs 4A and 5A show the MS/MS spectra of the linear octapeptide at m/z 1247.72 and of the nonapeptide at m/z 1410.78, respectively. All of the product ions obtained correspond with the peptide sequences of the putative linear octapeptide (Glu-Orn-Tyr-Thr-Glu-Val-Pro-Gln) and nonapeptide (Glu-Orn-Tyr-Thr-Glu-Val-Pro-Gln-Tyr). This result demonstrated that the matched linker (i.e. the native T-TE linker) could induce the TE domain to catalyse the hydrolytic cleavage of the mature linear depsipeptides from the hybrid peptide synthetases.

Moreover, we detected these ions ([M + H]^+^ 1087.6, 1101.6, 1115.7, 1129.7 m/z) in the crude extracts of the recombinant strain LP4, with a mass difference of 18 Da from the characterized linear heptapeptides (Table 2), suggesting that these are the cyclic forms of the corresponding linear heptapeptide ions with a loss of H_2_O. Further support for this result was obtained from analysis of the ESI-MS/MS spectrum of [M + H]^+^ ions at 1101.65 m/z, as shown in Fig. 3B. The cyclic molecule fragments yielded characteristic product ions –y_5_ (590.28) and –y_6_ (704.36), corresponding to the macrocyclic moiety and the macrocycle along with the Orn residue, respectively. Similarly, cyclic octapeptides ([M + H]^+^ 1229.7, 1243.7, 1257.7 m/z) were also observed in the crude extracts of the recombinant strains LP5 and LP6, which were further characterized by MS/MS (Fig. 4B). The LC-MS/MS data revealed that lactone ring formation occurred between the hydroxyl group of l-Tyr3 and the carboxy terminus of l-Pro7 in the cyclic heptapeptide, or in the carboxy terminus of l-Gln8 in the cyclic octapeptide.

## Discussion

Peptide synthetases are encoded by genes organized in a modular structure, in which repeated domains are associated with specific functions. This organization in structurally and functionally separated regions suggests that the order and type of building units can be genetically altered to create new enzymes with novel specificity and to produce novel peptides. However, to date, there has been limited success in producing novel compounds by manipulation of NRPSs using a domain or module exchange strategy^10^,^11^,^13^,^24^. Recent studies have focused on the *in vitro* characterization of several TE domains using purified proteins, including bimodular NRPSs. Using an *in vitro* approach, rationally designed cyclic peptides and antibiotics were synthesized via TE-catalysed chemoenzymatic biosynthesis^18^,^25^,^26^.

We have previously shown that maintenance of the C-terminal TE function in the plipastatin synthetase complex is necessary for efficient plipastatin production. Although deletion of the TE domain had a negative influence on plipastatin production, the unmatched linker region between the TE domain and various T domains from other modules rendered the hybrid enzyme inactive, even in the presence of normal amounts of the truncated enzyme. A previous study of the gramicidin TE domain showed that the catalytic efficiency of GrsTE_short_ was 5-fold lower than that of GrsTE_long_ *in vitro*, indicating an important role of the N-terminal extension for protein stability^18^. Accordingly, we fused the TE_long_ domain of plipastatin synthetase to the end of different T domains in the PPSD subunit. These newly derived hybrid enzyme complexes could all successfully produce the peptides efficiently. The expected peptides were detected from the NRPS-engineered strains, suggesting that the native T-TE linker is necessary for the catalytic activity of the TE domain, and that a T-C or T-E linker in truncated peptide synthetase would render it non-functional. Thus, we hypothesized that the T-TE linker, and by analogy the inter-domain linker, may not simply act as a tether to ensure that the domains are aligned and in close proximity but also ensures a certain specificity between various domains. Thus, the T-TE linker may serve as a tool for efficient communication between the T domain and TE domain. Structural analysis of the T and TE domains might provide further insight into these mechanistic processes.

In the engineered strains LP4, LP5, and LP6, the plipastatin TE domain could use water as an attracting nucleophile to catalyse the cleavage of the mature product, thus leading to the generation of linear hepta-, octa-, and nonapeptides. This observation proved that the plipastatin TE domain has hydrolytic activity and does not show strict substrate specificity, instead acting as a hydrolase in the truncated hybrid enzyme complex. Similar findings have been reported with respect to surfactin and tyrocidine NRPSs; in both cases, the TE domain was fused to other modules, and the resulting enzyme could only catalyse the hydrolysis of predicted peptides^4^,^23^. In contrast, the plipastatin TE domain not only catalysed the hydrolysis of the peptides but also catalysed the products’ cyclization. The cyclic hepta- and octapeptides were respectively detected in the mutant strains LP4 and LP5, which clearly indicated that the plipastatin TE domain could recognize the last amino acid, Pro or Gln, and catalyse it directly to participate in the cyclization reaction. Furthermore, this finding indicates the relatively relaxed substrate specificity of the plipastatin TE domain for macrocyclization. Plipastatin, are almost identical fengycin. Stefan A. Samel *et al*.^16^ reported that FenTE shares 32% to 38% sequence identity with the thioesterase domains from NRPS. In contrast, there is a mere 17% sequence identity with the 6-deoxyerythronolide TE from polyketide synthases (PKS). In addition, these studies about NRPS-derived TE domains^15^,^16^,^27^,^28^ all suggested that TE domain recognized only the last few residues at the C-terminus and the residues near the attacking nucleophile during the peptide cyclization *in vitro*. Our vivo experiment results are also consistent with this finding that the _L_-Pro7, _L_-Gln8 and _L_-Tyr9 are near the active site canyon in the model of the FenTE-fengycin complex^16^.

Plipastatin TE_long_ was fused into the end of the ninth T domain, which generated a truncated plipastatin enzyme complex composed of nine modules in the mutant strain LP6. We not only detected the expected linear nonapeptide but also detected both linear and cyclic octapeptides. These results imply that the ninth module could be skipped during the assembly process. Module skipping has recently been described for an engineered PKS system, and the skipping process was shown to involve passage of the growing polyketide through the skipped module by direct acyl carrier protein (ACP)-to ACP transfer^29^. To our knowledge, there has been only one example reported to date describing module skipping during an NRPS-catalysed assembly process. In this case, the growing peptide chain was directly transferred from module 3 to module 5^30^, which differs from the PKS system, in which the interpolated ACP is required for the skipping process^29^. In this context, this new hybrid biosynthetic system appears to be more flexible than the original complex, so that the precursor octapeptide chain was not only transferred to the adjacent module 9 to generate linear nonapeptides but was also transferred directly to the TE domain to generate both linear and cyclic octapeptides. This represents the first demonstration that module skipping can be achieved by simply moving the TE domain forward in an NRPS system.

The yield of original plipastatin produced by *B. subtilis* pB2-L was only 10 mg/L, which may be result in the production level of derivatives in these experiments were very low. Similar yield reduction has been reported in many engineering NRPS. For example, the production of novel antibiotics related to daptomycin generally ranged from about 1% to 50% of control, which were generated by module exchanges, NRPS subunit exchanges, inactivation of the tailoring enzyme and natural variations of the lipid tail^3^,^13^. The yields of surfactin derivatives generated by modified the surfactin synthetase were also relatively low^10^,^11^,^23^,^31^. Lipopeptides produced from the engineering synthetases can be interesting for pharmaceutical applications, even though the reduction compared with the wild-type enzyme. It is possible that this approach could be applied to industrial plipastatin production for generating significant quantities of plipastatin derivatives with improved biological activities. Besides, we might anticipate improving the yields by modification of the regulatory regions and optimizing fermentation parameters.

In summary, our approach enables the construction of truncated hybrid enzymes to synthesize peptides of desired length and amino acid composition, and provides important insights into the regioselectivity of plipastatin TE, which could be used to rationally manipulate the ring size of macrocyclic product. We believe that the plipastatin TE domain has great potential in the engineering of peptide synthetases for generating many new analogues of active peptides, especially non-linear and modified peptides that are difficult and costly to produce via chemical synthesis.

## Additional Information

**How to cite this article**: Gao, L. *et al*. Translocation of the thioesterase domain for the redesign of plipastatin synthetase. *Sci. Rep.* **6**, 38467; doi: 10.1038/srep38467 (2016).

**Publisher's note:** Springer Nature remains neutral with regard to jurisdictional claims in published maps and institutional affiliations.

## Supplementary Material

Supplemental Table S1

## Figures and Tables

**Table 1:** Strains and plasmids.

Strain or plasmid	Relevant characteristic^a^	Reference or source
*Bacillus subtilis*		
pB2	*B. subtilis* 168 derivative, laboratory strain; *trpC degQ*^0^ *sfp*^0^; Srf– Pps–	Chester Price’ lab (UCDavis, USA)
pB2-L	*B. subtilis* pB2 derivative with a P43-sfp^+^-degQ^+^ cassette inserted at the *amyE* locus; producing plipastain (Pps^+^) and surfactin (Srf^+^); Cm^R^	Constructed and characterized in our lab, stored in CGMCC (No. 11723)
LP1	*B. subtilis* pB2-L derivative with TE domain fused at end of the 7^th^ T domain; Pps− Srf^+^ Cm^R^	This study
LP2	*B. subtilis* pB2-L derivative with TE domain fused at end of the 8^th^ T domain; Pps− Srf^+^ Cm^R^	This study
LP3	*B. subtilis* pB2-L derivative with TE domain fused at end of the 9^th^ T domain; Pps− Srf^+^ Cm^R^	This study
LP4	*B. subtilis* pB2-L derivative with TE_Long_ domain fused at end of the 7^th^ T domain; producing heptapeptide; Pps− Srf^+^ Cm^R^	This study
LP5	*B. subtilis* pB2-L derivative with TE_Long_ domain fused at end of the 8^th^ T domain; producing octapeptide; Pps− Srf^+^ Cm^R^	This study
LP6	*B. subtilis* pB2-L derivative with TE_Long_ domain fused at end of the 9^th^ T domain; producing octapeptide and nonapeptide; Pps− Srf^+^ Cm^R^	This study
*E. coli*		
DH5α	*recA1*, *endA1*, *lacZ*ΔM15	Vazyme (Nanjing, China)
JM110	F’,*traD36 proA*^*+*^*B*^*+*^ *lacI*^*q*^ *lacZ*ΔM15/*dam dcm supE44 hsdR17 thi leu thr rpsL lacY galK galT ara tonA tsx*Δ (lac-proAB)	Transgen Biolabs (Beijing, China)
Plasmids		
pMD19T-simple	TA cloning vector; Ap^R^	TaKaRa (Dalian, China)
pKS2	Thermosensitive vector; Kan^R^, Erm^R^	22
pKS-7ProTTE	7ProTlinker-TE fragment inserted into pKS2, Kan^R^, Erm^R^	This study
pKS-8GlnTTE	8GlnTlinker-TE fragment inserted into pKS2, Kan^R^, Erm^R^	This study
pKS-9TyrTTE	9TyrTlinker-TE fragment inserted into pKS2, Kan^R^, Erm^R^	This study
pKS-7ProTE_Long_	7ProTTE_Long_ fragment inserted into pKS2, Kan^R^, Erm^R^	This study
pKS-8GlnTE_Long_	8GlnTTE_Long_ fragment inserted into pKS2, Kan^R^, Erm^R^	This study
pKS-9TyrTE_Long_	9TyrTTE_Long_ fragment inserted into pKS2, Kan^R^, Erm^R^	This study

^a^Cm^R^, Ap^R^, Kan^R^, Erm^R^: resistant to chloramphenicol, ampicillin, kanamycin, and erythromycin, respectively.

Srf– Pps–: unable to synthesize either surfactin or plipastatin.

**Table 2:** Truncated lipopeptides produced by recombinant *B. subtilis* strains.

Strain	Mass ions detected by LC-MS	Peptide sequence	Products
pB2-L	1435.8, 1449.8, 1463.8, 1477.8, 1491.8, 1505.8, 1519.8, 1533.8	(C_14–21_)β-OHFA-E-O-Cyclo(Y-T-E-A/V-P-Q-Y-I/V)	Plipastatin
LP4	1105.6, 1119.6, 1133.7, 1147.7	Linear (C_16–19_)β-OHFA-E-O-Y-T-E-V-P	Linear heptapeptides
	1087.6, 1101.6, 1115.7, 1129.7	(C_16–19_)β-OHFA-E-O-cyclo(Y-T-E-V-P)	Cyclic heptapeptides
LP5	1233.7, 1247.7	Linear (C_16–17_)β-OHFA-E-O-Y-T-E-V-P-Q	Linear octapetides
	1229.7, 1243.7, 1257.7	(C_17–19_)β-OHFA-E-O-cyclo(Y-T-E-V-P-Q)	Cyclic octapeptides
LP6	1382.7, 1396.8, 1410.8	Linear (C_15–17_)β-OHFA-E-O-Y-T-E-V-P-Q-Y	Linear nonapeptides
	1219.7, 1233.7, 1247.7	Linear (C_15–17_)β-OHFA-E-O-Y-T-E-V-P-Q	Linear octapeptides
	1215.7, 1229.7, 1243.7, 1257.7	(C_16–19_)β-OHFA-E-O-cyclo(Y-T-E-V-P-Q)	Cyclic octapeptides
