# Supplementary material for: Translocation of the thioesterase domain for the redesign of plipastatin synthetase
Source: Sci Rep. 2016 Dec 23;6:38467. doi: 10.1038/srep38467 (PMC5180189; doi:10.1038/srep38467)
Supplement: Supplemental Table S1 [file srep38467-s1.doc]

# Translocation of the thioesterase domain for the redesign of plipastatin synthetase

Ling Gao^1^, Hongxia Liu^1^, Zhi Ma^1^, Jinzhi Han^1^, Zhaoxin Lu,^1^ Chen Dai^2^, Fengxia Lv^1^ & Xiaomei Bie*^1^

^1^College of Food Science and Technology, Nanjing Agricultural University, Key Laboratory of Food Processing and Quality Control, Ministry of Agriculture of China, 1 Weigang Nanjing 210095, P.R. China

^2^College of Life Science, Nanjing Agricultural University, Ministry of Agriculture of China, 1 Weigang Nanjing 210095, P.R. China

***Corresponding author:**

Xiaomei Bie

College of Food Science and Technology, Nanjing Agricultural University, Key Laboratory of Food Processing and Quality Control, Ministry of Agriculture of China, Nanjing, P.R. China

Tel: 0086-25-84396570; Fax: 0086-25-84396583; E-mail: bxm43@njau.edu.cn

**Supplemental Table S1.** PCR primers used for genetic constructs

| **Primers** | **The sequence (5′ to 3′)** | **Restriction sites** |
| --- | --- | --- |
| 7ProTlinker-F | GTCGACAACATCGAATATATCGGACGGG | *Sal*I |
| 7ProTlinker -R | TTCTGCTGGTTCAATTGCTGCAT |  |
| 8GlnTlinker-F | GTCGACAGCGGGTGTTGCCAGAGGTTATTTG | *Sal*I |
| 8GlnTlinker -R | TTCAGCGGGCTTAATCGCTTCATAT |  |
| 9TryTlinker-F | GTCGACAAACCCGAAGCACGAATGTAC | *Sal*I |
| 9TryTlinker -R | CGCAGCTTGGCTGGAACTTG |  |
| TE -F | CAAAATCTTTTCTGCTTCCC |  |
| TE -R | GGTACCGCAGTATTACTTGATTTGAAGTGA | *Kpn*I |
| P7-F | ATGCAGCAATTGAACCAGCAGAACAAAATCTTTTCTGCTTCCC |  |
| P7-R | GGGAAGCAGAAAAGATTTTGTTCTGCTGGTTCAATTGCTGCAT |  |
| P8-F | ATATGAAGCGATTAAGCCCGCTGAACAAAATCTTTTCTGCTTCCC |  |
| P8-R | GGGAAGCAGAAAAGATTTTGTTCAGCGGGCTTAATCGCTTCATAT |  |
| P9-F | CAAGTTCCAGCCAAGCTGCGCAAAATCTTTTCTGCTTCCC |  |
| P9-R | GGGAAGCAGAAAAGATTTTGCGCAGCTTGGCTGGAACTTG |  |
| 7ProT-F | GTCGACTACCATTCATCCTGGAGACC | *Sal*I |
| 7ProT-R | AGCTAATCCTTCAACAGTCG |  |
| 8GlnT-F | GTCGACAAGACAAACAGACTCAAGCGA | *Sal*I |
| 8GlnT-R | GATAACGGTAGCCAGTCCTTC |  |
| 9TyrT-F | GTCGACCCAACGAATATGGACCGACA | *Sal*I |
| 9TyrT-R | GATATAAGCTGCCAATTCTTGAATG |  |
| TE_Long_-F | TATTCTAAACAGCTGACAGC |  |
| TE_Long_-R | GGTACCTTATCGTTTGTGCAGTATTAC | *Kpn*I |
| S7-F | CGACTGTTGAAGGATTAGCTTATTCTAAACAGCTGACAGC |  |
| S7-R | GCTGTCAGCTGTTTAGAATAAGCTAATCCTTCAACAGTCG |  |
| S8-F | GAAGGACTGGCTACCGTTATCTATTCTAAACAGCTGACAGC |  |
| S8-R | GCTGTCAGCTGTTTAGAATAGATAACGGTAGCCAGTCCTTC |  |
| S9-F | CATTCAAGAATTGGCAGCTTATATCTATTCTAAACAGCTGACAGC |  |
| S9-R | GCTGTCAGCTGTTTAGAATAGATATAAGCTGCCAATTCTTGAATG |  |
